# Selenoprotein P, Peroxiredoxin-5, Renalase and Selected Cardiovascular Consequences Tested in Ambulatory Blood Pressure Monitoring and Echocardiography

**DOI:** 10.3390/antiox12061187

**Published:** 2023-05-30

**Authors:** Karolina Czerwińska, Lidia Januszewska, Iwona Markiewicz-Górka, Aleksandra Jaremków, Helena Martynowicz, Krystyna Pawlas, Grzegorz Mazur, Rafał Poręba, Paweł Gać

**Affiliations:** 1 Department of Population Health, Division of Environmental Health and Occupational Medicine, Wroclaw Medical University, Mikulicza-Radeckiego 7, PL 50-368 Wroclaw, Polandiwona.markiewicz-gó; 2 Department of Internal Medicine, Occupational Diseases, Hypertension and Clinical Oncology, Wroclaw Medical University, Borowska 213, PL 50-556 Wroclaw, Poland

**Keywords:** selenoprotein P, renalase, peroxiredoxin-5, OSA, ABPM, ECHO

## Abstract

This study aimed to assess the relationship between chosen antioxidants, namely selenoprotein P (SELENOP), peroxiredoxin-5 (Prdx-5), renalase and selected cardiovascular consequences tested in ambulatory blood pressure monitoring (ABPM) and echocardiography (ECHO). In our work, cardiovascular consequences refer to higher mean blood pressure (MBP) and pulse pressure (PP) on ABPM, as well as to left atrial enlargement (LAE), left ventricular hypertrophy (LVH) and lower left ventricular ejection fraction (LVEF%) on ECHO. The study group consisted of 101 consecutive patients admitted to the Department of Internal Medicine, Occupational Diseases and Hypertension to verify the diagnosis of Obstructive Sleep Apnoea (OSA). Each patient underwent full polysomnography, blood tests, ABPM and ECHO. Both selenoprotein-P and renalase levels correlated with different ABPM and ECHO parameters. We found no correlation between the peroxiredoxin-5 level and none of the tested parameters. We point to the possible application of SELENOP plasma-level testing in the initial selection of high cardiovascular-risk patients, especially if access to more advanced examinations is limited. We further suggest SELENOP measurement as a possible indicator of patients at increased left ventricular hypertrophy risk who should be of particular interest and may benefit from ECHO testing.

## 1. Introduction

Increased oxidative stress is a major contributing factor in cardiovascular disease (CVD) pathogenesis. Disbalance between reactive oxygen species (ROS) and antioxidants leads to a decrease in the bioavailability of nitric oxide (NO), endothelial dysfunction, and damage to numerous cellular structures. Therefore, antioxidants are often proposed as promising agents in both the diagnosis and therapy of CVDs. Despite growing evidence supporting the role of antioxidants in CVD pathogenesis, the measurement of antioxidants is not officially recommended for initial CVD diagnosis or cardiovascular risk assessment.

Selenoprotein P (SELENOP) belongs to a group of selenocysteine-containing selenoproteins, along with glutathione peroxidases (GPx), thioredoxin reductases (TrxR) and iodothyronine deiodinases (DIO) [1]. It is mainly produced in the liver and subsequently excreted into the plasma. SELENOP, unlike other selenoproteins, contains 10 selenocysteine (Sec) residues, which constitute a substantial selenium store. One N-terminal Sec residue forms an active site of enzyme activity to reduce phospholipid hydroperoxide, while the nine C-terminal Sec residues function as Se transporters to extrahepatic tissues [2,3]. To date, three kinds of SELENOP receptors have been identified, namely, ApoER2 (LRP8), megalin (LRP2), and LRP1. 3 After entering the cell, SELENOP is degraded to amino acids in the lysosome [3]. According to in vitro studies, SELENOP may act as a phospholipid hydroperoxide-GPx and as a peroxinitrite reductase [4]. Moreover, SELENOP-pretreated cells were protected from oxidative damage induced by tert-butyl hydroperoxide through an elevated biosynthesis of intracellular selenoenzymes [5]. Nevertheless, the exact mechanism of SELENOP involvement in redox balance remains unknown. Studies on SELENOP knockout (KO) mice underline the essential role of SELENOP in delivering Se to the brain and testes; however, the data on SELENOP significance in cardiovascular disease are scarce. Recent studies point to its possible involvement in the pathogenesis of Pulmonary Arterial Hypertension (PAH) [6]. It has been reported that serum levels of SeP were significantly higher in patients with PAH in comparison with control subjects. Moreover, patients with higher levels of SeP showed poorer prognoses in comparison with those with lower SeP levels. However, additional research is needed to understand the underlying mechanism behind the described correlation.

Renalase is a flavin adenine dinucleotide-dependent amine oxidase that serves as a scavenger enzyme. It oxidizes isomeric forms of β-NAD(P)H and recycles them by forming β-NAD(P)+. This action protects cells from the accumulation of β-NAD(P)H isomers that may inhibit other enzymatic reactions [7]. The importance of renalase’s enzymatic activity, as well as renalase’s involvement in intercellular signalling pathways, still remains poorly understood. It is yet to be discovered which enzymes are prone to inhibition by isomeric forms of β-NAD(P)H and what consequences this may have for the functioning of the cell. In our recent study, we found a positive linear relationship between the renalase blood concentration and the total antioxidant status (TAS) level, which seemed to confirm its antioxidant properties. Moreover, higher renalase levels were independently associated with TAS on regression analysis. We have summarised the latest reports on renalase’s enzymatic and non-enzymatic activity in our previous work [8].

Peroxiredoxins (Prdxs) are a family of peroxidases that maintain thiol homeostasis by catalysing the reduction of organic hydroperoxides, H_2_O_2_, and peroxinitrite [9]. Various experimental studies have confirmed the potential of Prdxs as a therapeutic strategy against CVD [9,10,11]. Moreover, it was suggested that the development of derivatives or mimetics of the catalytic activity of Prdxs offers great promise for antioxidant therapy in CVD [10].

Twenty four-hour ambulatory blood pressure monitoring (ABPM) is a method to measure blood pressure continuously. According to the American and European Guidelines on hypertension diagnosis and treatment, it is a preferable method to confirm office hypertension [12,13]. ABPM provides more reliable measurements and it helps to recognize masked and white coat hypertension. Moreover, increased blood pressure and pulse pressure on ABPM were found to correlate with the likelihood of cardiovascular and cerebrovascular disease and organ damage [14]. Dechen Liu et al. investigated whether PP was associated with all-cause and cause-specific mortality in a rural Chinese population. The study demonstrated that the risk of all-cause and other causes of mortality increased with increasing PP [15]. Pulse pressure was also described to predict left ventricular remodelling and left ventricular hypertrophy, particularly of the concentric type that is a significant independent risk factor for increased cardiovascular morbidity and mortality [16,17]. The use of ABPM is beneficial; however, it entails the availability of special equipment, knowledgeable personnel and time for interpretation, and thus, its accessibility may be limited in the outpatient care settings.

Echocardiography is an essential tool in the diagnosis, assessment and management of patients with clinical signs or symptoms of heart disease. Among hypertensive patients, it serves as a meaningful test in screening for specific signs of hypertensive heart disease, namely LV hypertrophy, LV diastolic dysfunction and LA enlargement [18]. These changes in heart morphology and function promote cardiovascular morbidity and mortality [19,20]. It is worth noting that concentric hypertrophy is related to the highest mortality and morbidity compared with other types of left ventricular geometry [21,22]. The concentric type of hypertrophy is characterised by elevated relative wall thickness (RWT) and increased mass indexed to body surface area (LVMI, g/m^2^) both measured during ECHO. While ECHO is highly useful, it is often not accessible in outpatient settings. In general, ECHO is recommended for hypertensive patients suspected of left ventricular hypertrophy. The office assessment of LVH may be difficult, and numerous ECG criteria may be applied; however, they differ in accuracy and have quite a low sensitivity of less than 50% [23]. For patients who have LVH but do not meet the ECG-LVH criteria, other predictive factors/clinical tests are being searched for [24].

Obstructive sleep apnoea (OSA) is a common respiratory sleep disorder characterized by recurrent partial (hypopnea) or complete (apnoea) obstruction of the upper airway during sleep, which results in intermittent hypoxia, arousals and sleep fragmentation [25,26]. Polysomnography (PSG) is the gold standard for OSA diagnosis. The PSG recording includes electroencephalography, electromyography, electrooculography, pulse oximetry, electrocardiography and a microphone. The parameter that defines the severity of OSA is the apnoea/hypopnoea index (AHI), which indicates the number of episodes per hour. OSA is diagnosed if the AHI is ≥ 5 events per hour [25]. OSA is a recognized cause of secondary hypertension. It is independently associated with cardiovascular morbidity and mortality [27]. Disturbed sleep physiology with repeated episodes of apnoea results in sympathetic over-activation and severe inflammation, which is considered a significant cause of increased cardiovascular risk in this patient group [28].

The aim of this study was to assess the relationship between chosen antioxidants, namely selenoprotein P (SELENOP), peroxiredoxin-5 (Prdx5), renalase and selected cardiovascular consequences tested using ambulatory blood pressure monitoring (ABPM) and echocardiography (ECHO). In our work, cardiovascular consequences refer to a higher mean blood pressure (MBP) and pulse pressure (PP) on ABPM, as well as to left atrial enlargement (LAE), left ventricular hypertrophy (LVH) and a lower left ventricular ejection fraction (LVEF%) on ECHO.

## 2. Materials and Methods

The research group consisted of 112 consecutive patients admitted to an internal medicine clinic to verify the diagnosis of OSA. The inclusion criteria for the study were as follows: consent to participate in the study and age ≥ 18 years. The exclusion criteria included the coexistence of severe systemic diseases, severe mental illness/mental disorders, and active proliferative disease. The characteristics of the study group with basic anthropometric measurements, and the diagnosis of OSA or hypertension (HTN), hypotensive treatment and mean blood selenoprotein P, renalase and peroxiredoxin-5 level are presented in Table 1.

For detailed analysis, the patients were divided into subgroups. In total, 20 subgroups (A–U) were distinguished. The division was made on the basis of the diagnosis of arterial hypertension (subgroups A and B), the diagnosis of obstructive sleep apnoea (subgroups C and D), the median level of selenoprotein P (subgroup E and F), the median level of peroxiredoxin-5 (subgroup G and H), the median level of renalase (subgroup I and J), the median of the mean blood pressure (subgroup K and L), the median pulse pressure (subgroup M and N), the median left atrium diameter (subgroup O and P), the median left ventricular ejection fraction (subgroup R and S), and the diagnosis of left ventricular hypertrophy (subgroup T and U). All selected subgroups are summarised in Table 2.

PSG was performed in the Sleep Laboratory of the Department of Internal Medicine, Occupational Diseases, Hypertension and Clinical Oncology, Wroclaw Medical University, Poland, according to a diagnostic standard as a nocturnal, single-night recording, using the Nox-A1 machine (Nox Medical, Reykjavík, Iceland). Polysomnograms were assessed in 30 s epochs according to the American Academy of Sleep Medicine (AASM) 2013 standard criteria for sleep scoring. The analysis of the collected data was performed by a qualified physician (H.M.) from the Sleep Laboratory. The PSG recordings included electroencephalography, electromyography, electrooculography, pulse oximetry, electrocardiography and a microphone. The airflow was measured using a nasal pressure transducer, and the respiratory effort of thoracoabdominal movement was measured using respiratory inductance plethysmography. The AHI was defined as the average number of episodes of apnoea and hypopnea per hour of total sleep time (TST). Apnoea was attained with the reduction of airflow to less than 10% of the baseline for at least 10 s. A hypopnea episode was defined as a decrease in the nasal pressure signal by at least 30% from baseline for at least 10 s, with a reduction in O_2_ saturation of at least 3% from the pre-event baseline or arousal.

Blood was collected in the morning after polysomnography, usually by puncturing the veins of the ulna. Until renalase determinations were performed simultaneously in all samples, the blood was stored at a constant temperature. Serum renalase determinations were performed using the E3109Hu kit ELISA (enzyme-linked immunosorbent assay) (Bioassay Technology Laboratory, Shanghai, China). The determinations were made strictly according to the test manufacturer’s instructions. The renalase concentration was expressed as nanogram per millilitre (ng/mL). The reference range of the assay used was 1–400 ng/mL. According to the manufacturer, the sensitivity of the ELISA test used was 0.52 ng/mL. The coefficient of intra- and inter-assay variation was <8% and <10%, respectively.

Serum selenoprotein P determinations were performed using the E1809h ELISA Kit for Human SeP (ElAab, East Lake Hi-Tech Development Zone, Wuhan, China). The determinations were made strictly according to the test manufacturer’s instructions. The selenoprotein P concentration was expressed as a nanogram per millilitre (ng/mL). The coefficient of intra- and inter-assay variation was <4.9% and <7.1%, respectively.

Serum peroxiredoxin-5 determinations were performed using the E0703h ELISA Kit for Human Peroxiredoxin-5, mitochondrial (ElAab, East Lake Hi-Tech Development Zone, Wuhan, China). The determinations were made strictly according to the test manufacturer’s instructions. The peroxiredoxin-5 concentration was expressed as a nanogram per millilitre (ng/mL). The reference range of the assay used was 0.78–50 ng/mL.

In every examined individual, 24 h ambulatory blood pressure monitoring was performed using the Welch Allyn ABPM 6100 system (Welch Allyn, UK, Aston Abbotts, Buckinghamshire, UK). The studied variables included the mean blood pressure (MBP), mean systolic blood pressure (MSBP), mean diastolic blood pressure (MDBP), variability of systolic blood pressure (VSBP), variability of diastolic blood pressure (VDBP) and pulse pressure (PP). Pulse pressure was calculated as the difference between MSBP and MDBP. Standard deviation (SD) from all measurements of systolic/diastolic blood pressure, taken at 30 min intervals, was accepted as a measure of VSBP and VDBP.

Transthoracic echocardiography was performed using the ALOKA ProSound SSD-5500 SV, equipped with a 3.5/2.7 MHz transducer (Aloka Inc, Tokyo, Japan). The results were evaluated using the criteria of the American Society of Echocardiography (ACC/AHA, 1990). Using an M-mode echocardiogram, following Penn convention, left ventricular end-diastolic diameter (LVEDd) and left ventricular end-systolic diameter (LVESd), interventricular septum diastolic diameter (IVSDd) and posterior wall diastolic diameter (PWDd) were measured. The mean of three measurements was recorded with an accuracy of 1 mm. The ejection fraction (EF) was determined from the apical four-chamber and two-chamber views, with the biplane Simpson’s method. The left ventricular mass (LVM), expressed in grams, was calculated using the formula suggested by the American Society of Echocardiography (ASE), modified by Devereux et al. (1986): LVM = 0.8 × [1.04 × (LVEDd+ PWDd+ IVSDd)^3^ − LVEDd^3^] + 0.6 (LVEDd, PWDd, and IVSDd expressed in centimetres). The left ventricular mass index (LVMI) was calculated, by dividing the LVM value by the body surface area (BSA), expressed in square meters. The body surface area was calculated using the formula of Du Bois: BSA [m^2^] = 0.007184 × (body weight [kg]) 0.425 × (body height [cm]) 0.725. The relative wall thickness (RWT) was calculated using the following formula: RWT = (IVSDd + PWDd)/LVEDd. The results of ABPM and ECHO in the study group are presented in Table 3.

Statistical analyses were conducted using the Dell Statistica 13 software (Dell Inc., Round Rock, TX, USA). The quantitative variables were expressed as means and standard deviations. The qualitative variables were expressed as percentages. The distribution of variables was tested with the W-Shapiro–Wilk test. In the case of the quantitative variables of normal distribution, a further statistical analysis was performed using the *t*-test. For non-normally distributed quantitative variables, the Mann–Whitney U test was used. For qualitative variables, the chi-square test of maximum likelihood was used. To determine the relationship between the examined variables, correlation and regression analyses were conducted. In the case of normal distribution, the Pearson correlation r factors were determined whereas, in the case of non-normal distribution, the Spearman r factors were applied. Multivariable stepwise backward regression was used to identify possible predictor variables for pulse pressure, left atrium diameter and left ventricular hypertrophy. Three separate analyses were performed with PP, LA and LVH as dependent variables. At each step, independent variables were removed from the model based on *p*-values. Results with *p* < 0.05 were considered statistically significant.

Ethical approval for this study was obtained from the Bioethics Committee of Wroclaw Medical University. Informed consent was obtained from all subjects before the study.

ClinicalTrials.gov Identifier: NCT05040516.

## 3. Results

In our study, patients with diagnosed hypertension (subgroup A) had statistically significantly lower selenoprotein P levels than patients without HT (subgroup B). Levels of peroxiredoxin-5 and renalase did not differ significantly between these groups. In terms of ABPM/ECHO parameters, patients with elevated blood pressure had significantly higher MBP, PP, and LA diameter and were more likely to have left ventricular hypertrophy. These results are shown in Table 4.

When dividing patients by the diagnosis of OSA (subgroup C and D), we did not find any significant differences in selenoprotein P, peroxiredoxin-5 and renalase levels, as well as in MBP values, LA diameter and the occurrence of LVH. However, patients with OSA (subgroup C) had significantly higher PP and the possibility of LVH. These results are shown in Table 4.

Comparison of results based on the criteria of median selenoprotein P, peroxiredoxin-5 and renalase levels showed that patients with selenoprotein P level ≥ median (subgroup E) had lower MBP, PP and LA diameter values and were less likely to develop LVH than patients with selenoprotein P level < median (subgroup F). None of the considered parameters differed significantly between patients with peroxiredoxin level ≥ median (subgroup G) and < median (subgroup H). Division by the median of renalase disclosed significantly lower PP and LA diameter values in patients with renalase level ≥ median (subgroup I) than in the subgroup with lower renalase levels (subgroup J); there were no differences in MBP, LVEF and LVH incidence between these subgroups. The analysis of selected ABPM/ECHO parameters based on the division by laboratory parameters is presented in Table 5.

No differences in laboratory parameters (SELENOP, peroxiredoxin-5 and renalase) levels were found when dividing patients by the median of MBP (subgroup K and L). However, both selenoprotein P and renalase levels were notably lower in patients with PP value ≥ the median (subgroup M) than in patients with lower PP values (subgroup N). Division by the median of MBP showed no differences between these subgroups in terms of LVH incidence. On the contrary, patients with higher PP (subgroup M) had a notably higher incidence of LVH than patients with PP lower than the median (subgroup N). These results are shown in Table 6.

Analysis based on selected ECHO parameters showed that selenoprotein P and renalase levels were significantly lower in patients with LA diameter ≥ median (subgroup O) than in patients with LA diameter < Me, as well as in patients with diagnosed LVH (subgroup T) than in subjects without LVH (subgroup U). Patients with lower LVEF% (subgroup R) had markedly lower PP values than patients with LVEF% ≥ median (subgroup S). On the other hand, PP was significantly higher in patients with higher LA diameter (subgroup O) and in patients with LVH (subgroup T). None of the tested laboratory parameters differed significantly between people with lower or higher LVEF% (groups R vs. S). Analysis based on selected ECHO parameters is presented in Table 7.

In this study, negative linear correlation was found between selenoprotein P and certain ABPM and ECHO parameters, namely: MSBP (r = −0.33, *p* < 0.05), MDBP (r = −0.21, *p* < 0.05), MBP (r = −0.28), *p* < *0*.05), PP (r = −0.32, *p* < 0.05), IVSEDD (r = −0.28, *p* < 0.05), PWEDD (r = −0.27, *p* < 0.5), LA (r = −0.3, *p* < 0.05) and LVMI (r = −0.33, RWT (r = −0.2, *p* < 0.05). Renalase level correlated in a negative linear manner with PP (r = −0.22, *p* < *0*.05), IVSEDD (r = −0.2, *p* < 0.05) and LA (r = −0.22, *p* < 0.05). The results of correlation analysis are presented in Table 8.

A regression analysis was performed for three different dependent variables: PP, LA and LVH. A final model obtained for PP as a dependent variable was:

PP = 44.801–3.757 antihypertensive drugs–1.876 selenoprotein P–0.011 renalase + 0.118 age + 0.355 BMI

The obtained model demonstrated that no antihypertensive drug therapy, low selenoprotein P level, low renalase level, more advanced age and higher BMI are independent risk factors for elevated pulse pressure.

A final model obtained for LA as a dependent variable was:

LA = 38.929 + 0.045 age + 0.004 BMI + 1.762 male gender + 0.111 PP

Based on the obtained regression model, it was shown that more advanced age, higher BMI and male gender represented independent risk factors for greater left atrium diameter.

A final model obtained for LVH was:

P (LVH) = 0.079–1.448 antihypertensive drugs + 1.210 OSA + 0.043 PP

The obtained regression model indicated that no antihypertensive drug therapy, diagnosis of obstructive sleep apnoea and elevated pulse pressure represented independent risk factors for left ventricular hypertrophy.

**Figure 1:**
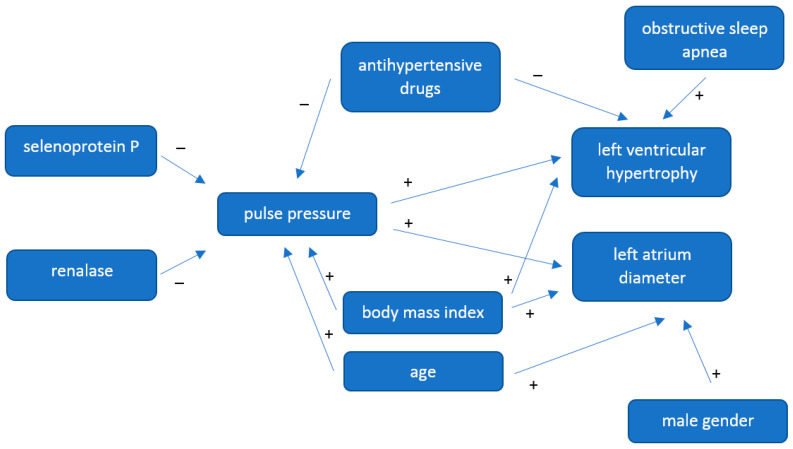
Diagram summarizing the regression analysis.

Detailed results of regression analysis in the study group are presented in Table 9. Figure 1 shows a diagram summarizing the results of the regression analysis. This section may be divided by subheadings. It should provide a concise and precise description of the experimental results, their interpretation, as well as the experimental conclusions that can be drawn.

## 4. Discussion

The key finding of this study is that both selenoprotein-P and renalase levels correlated significantly with different ABPM and ECHO parameters. We found no correlation between the peroxiredoxin-5 level and the tested parameters.

Peroxiredoxins (Prdxs) are a superfamily of selenium-free and haeme-free peroxidases able to catalyse the reduction of hydrogen peroxide, alkyl hydroperoxides and peroxinitrite [29]. In humans, the PRDXs family comprises six isoforms (Prdx1–6), of which all have been proposed to be involved in CVD pathogenesis [11]. Prdx 1, 2 and 4 were described to play protective roles in the development of atherosclerosis [11]. The overexpression of Prdx3 was found to protect the heart against left ventricular remodelling and failure after myocardial infarction. Prdx6-deficient mice had increased susceptibility to ischemia-reperfusion injury [11]. Prdx5 was the last family member to be identified. It is thought to exert antioxidative and cytoprotective properties; however, the exact mechanism of its function and its importance remain to be fully elucidated [30]. Hu C. et al. described that Prx5 expression was upregulated in hypertrophic hearts and cardiomyocytes. In addition, Prx5 knockdown accelerated pressure overload-induced cardiac hypertrophy and dysfunction in mice by activating oxidative stress and cardiomyocyte apoptosis [31]. Kunze A et al. reported on decreased Prdx5 levels in severe stroke [32]. In this study, we failed to find any significant correlations between PRDx5 plasma level and the tested parameters. Thus, at this point, we do not recommend the use of Prdx5 plasma level measurement to be used in selecting patients with an increased risk of cardiovascular complications in hypertensive patients.

In our recent work, we have reported that a higher SELENOP level was independently associated with higher plasma TAS, and TAS was further associated with lower mean blood pressure values. The obtained results suggested an indirect connection between the SELENOP level and blood pressure. In this study, we report that hypertensive patients had significantly lower SELENOP levels than normotensive patients (*p* < 0.05). In the light of our previous results, the association between SELENOP and mean blood pressure may possibly be explained by SELENOP’s involvement in maintaining the right plasma TAS and keeping the redox balance.

In terms of the ABPM parameters, patients with SELENOP levels higher than the median had a notably lower mean blood pressure and pulse pressure when compared to the patients with lower SELENOP levels. We also found a negative linear correlation between SELENOP and mean systolic blood pressure (r = −0.33, *p* < 0.05), mean diastolic blood pressure (r = −0.21, *p* < 0.05), mean blood pressure (r = −0.28), *p* < 0.05) and pulse pressure (r = −0.32, *p* < 0.05). Furthermore, regression analysis revealed that low SELENOP levels was an independent risk factor for higher PP with a *p*-value under 0.01. In sum, a lower SELENOP level was associated with unfavourable cardiovascular consequences measured on ABPM–higher MBP, MDBP, MSBP and PP. This result points to the possible applicability of SELENOP measurement in the initial selection of high cardiovascular-risk patients, especially if access to more advanced examinations is limited. Proper cardiovascular risk stratification remains a fundamental step in the effort to reduce morbidity and mortality from cardiovascular diseases (CVDs) [33]. With a constantly growing number of people suffering from CVDs, it is challenging to choose patients at the highest risk of premature death and to tailor the right treatment. Interestingly, our results stay in line with the findings of a study carried out by Schomburg et al. in which SELENOP deficiency predicted cardiovascular morbidity and mortality [34]. It was a population-based prospective cohort study, which included 4366 subjects during a median follow-up time of 9.3 (8.3–11) years. The 20% of subjects with the lowest SELENOP concentrations without a history of cardiovascular disease had markedly increased risk of cardiovascular morbidity and mortality. It is worth noting that the mean concentration of SELENOP within our study group was quite low and would be considered the lowest quantile from the Schomburg study.

In terms of ECHO parameters, we have found a negative linear correlation between SELENOP levels and both LVMI (r = −0.33) and RWT (r = 0.20). Moreover, patients with SELENOP levels higher than the median had notably lower left atrium diameter and were less likely to develop left ventricular hypertrophy (*p* < 0.05). Based on the information provided, we suggest SELENOP measurement as a possible indicator of patients at increased LVH risk who should be of particular interest and may benefit from ECHO testing. The application of SELENOP measurement in the risk stratification seems more achievable than aiming to increase its level with therapy. Selenium supplementation may be a promising strategy in preventing hypertension and its cardiovascular consequences; nevertheless, this issue is more complex. Data on selenium supplementation are inconclusive as both beneficial and harmful effects have been reported [35,36,37,38,39,40]. It is suggested that subjects with low selenium levels at baseline could benefit from supplementation; on the contrary, those with an adequate or high status might be negatively affected [40].

The notion that renalase serves to degrade catecholamines has been dismissed; however, many studies still point to its involvement in blood pressure regulation. The reports on this subject are inconclusive. Many studies indicate higher renalase concentration in hypertensive patients [41,42], and many studies show contrary results [43,44]. Our analysis revealed that patients with a renalase level ≥ median (subgroup I) had significantly lower PP and LA diameter values than patients in the subgroup with a lower renalase level (subgroup J). Moreover, renalase levels correlated in a linear negative manner with PP (r = −0.22, *p* < 0.05) and a low renalase level was an independent risk factor for higher pulse pressure in the regression analysis with a *p*-value under 0.05. We thus hypothesise that renalase may be more involved in keeping the balance between systolic and diastolic blood pressure rather than in regulating crude blood pressure values. However, taking into consideration the complexity of renalase activity and its enzymatic and non-enzymatic properties, many more studies are needed to elucidate this issue.

In our study, independent risk factors for LVH included a higher pulse pressure on ABPM, higher body mass index (BMI), lack of antihypertensive treatment and the diagnosis of obstructive sleep apnoea (OSA). Interestingly, mean blood pressure values were less effective in predicting LVH than pulse pressure; thus, we propose PP as the preferable parameter to be used in CV risk stratification and selecting patients who require further care and treatment. Independent risk factors for left atrium enlargement included higher PP and higher BMI, but also more advanced age and male gender. These results stay consistent with other studies on LA diameter, BMI and gender [45].

Obstructive sleep apnoea was an independent risk factor for LVH. It should be underlined that when dividing patients by the diagnosis of OSA (groups C and D), we did not find any differences in MBP values; however, patients with OSA had significantly higher PP. We indicate OSA patients as the group of special interest and recommend their PP to be checked even if blood pressure remains within the normal range. Interestingly SELENOP levels did not differ significantly between patients with and without OSA, suggesting that SELENOP may rather be involved in the pathomechanism of primary than secondary hypertension. Authors should discuss the results and how they can be interpreted from the perspective of previous studies and of the working hypotheses. The findings and their implications should be discussed in the broadest context possible. Future research directions may also be highlighted.

This study has some limitations. In terms of the study group, the limitations are the small size of the group and the relatively small percentage of patients without OSA and without abnormal body weight. In terms of the research methodology, the limitations of the ABPM analysis include the absence of separate measurements from the hours of daily activity and the measurements from the hours of night rest, the lack of assessment of the diastolic function of the left ventricle in the ECHO examination and the subjective selection of antioxidants.

## 5. Conclusions

In our study, both selenoprotein-P (SELENOP) and renalase levels correlated significantly with different ambulatory blood pressure monitoring (ABPM) and echocardiography (ECHO) parameters. We found no correlation between the peroxiredoxin-5 level and the tested parameters. A lower SELENOP level was associated with unfavourable cardiovascular consequences measured on ABPM–higher mean blood pressure, mean diastolic blood pressure, mean systolic blood pressure and pulse pressure, as well as on ECHO–increased left ventricle mass index and relative wall thickness. We point to the possible application of SELENOP plasma-level testing in the initial selection of high cardiovascular-risk patients, especially if access to more advanced examinations is limited. We further suggest SELENOP measurement as a possible indicator of patients at increased left ventricular hypertrophy risk who should be of particular interest and may benefit from ECHO testing. In this study group, renalase levels did not differ significantly between subjects with or without diagnosed hypertension. However, our analysis revealed that renalase level correlated in a linear negative manner with pulse pressure, and low renalase level was an independent risk factor for higher pulse pressure in the regression analysis. Given that reports on renalase are inconsistent, its clinical use appears to be delayed until its exact function is recognised.

## Figures and Tables

**Table 1:** Characteristics of the study group (*n* = 101).

Variable	Prevalence in the Study Group
age (years) ^a^ height (cm) ^a^ body mass (kg) ^a^ BMI (kg/m^2^) ^a^ BSA (m^2^) ^a^	51.06 ± 13.93 172.22 ± 10.16
	85.96 ± 15.36
	29.04 ± 5.05
	1.99 ± 0.20
HTN ^b^ sBP (mmHg) ^a^ dBP (mmHg) ^a^ diuretics ^b^ β-blockers ^b^ ACE inhibitors ^b^ angiotensin receptor blockers ^b^ calcium channel blockers ^b^	40.6
	139.90 ± 20.83
	89.95 ± 12.44
	17.8
	19.8
	18.8
	10.9
	8.9
OSA ^b^ mild OSA ^b^ moderate OSA ^b^ severe OSA ^b^ AHI (events/h) ^a^	75.2
	30.7
	20.8
	23.8
	18.34 ± 18.46
type 2 diabetes ^b^ coronary artery disease ^b^	8.9
	7.9
selenoprotein P (ng/mL) ^a^ peroxiredoxin-5 (ng/mL) ^a^ renalase (ng/mL) ^a^	1.50 ± 1.91
	1.48 ± 3.67
	178.56 ± 208.00

^a^—values represent mean ± standard deviation, ^b^—values represent percentages, AHI—apnoea-hypopnea index, BMI—body mass index, BSA—body surface area, dBP—diastolic blood pressure, HTN—arterial hypertension, OSA—obstructive sleep apnoea, sBP—systolic blood pressure.

**Table 2:** Criteria for selecting subgroups.

	Subgroup	Classification Criterion	Subgroup Size
division by HTN diagnosis	A	diagnosed with HTN	60
	B	without HTN	41
division by OSA diagnosis	C	diagnosed with OSA	76
	D	without OSA	25
division by median of selenoprotein P	E	≥median of selenoproteins P (≥0.64 ng/mL)	51
	F	<median of selenoproteins P (<0.64 ng/mL)	50
division by median of peroxiredoxin	G	≥median of peroxiredoxin (≥0.77 ng/mL)	52
	H	<median of peroxiredoxin (<0.77 ng/mL)	49
division by median of renalase	I	≥median of renalase (≥60.43 ng/mL)	51
	J	<median of renalase (<60.43 ng/mL)	50
division by median of MBP	K	≥median of MBP (≥93.47 ng/mL)	51
	L	<median of MBP (<93.47 ng/mL)	50
division by median of PP	M	≥median of PP (≥51.00 mmHg)	53
	N	<median of PP (<51.00 mmHg)	48
division by median of LA	O	≥median of LA (≥43.00 mm)	53
	P	<median of LA (<43.00 mm)	48
division by median of LVEF	R	≥median of LVEF (≥66%)	54
	S	<median of LVEF (<66%)	57
division by diagnosis of LVH	T	diagnosed LVH	64
	U	without LVH	37

HTN—arterial hypertension, LA—left atrium diameter, LVEF—left ventricular ejection fraction, LVH—left ventricular hypertrophy, MBP—mean blood pressure, OSA—obstructive sleep apnoea, PP—pulse pressure.

**Table 3:** Selected parameters of 24-h ambulatory blood pressure monitoring and echocardiography in the study group (*n* = 101).

Measured Parameter	Results
MSBP (mmHg) ^a^	131.57 ± 19.02
MDBP (mmHg) ^a^	76.70 ± 10.63
MBP (mmHg) ^a^	94.81 ± 12.68
VSBP (mmHg) ^a^	13.78 ± 4.19
VDBP (mmHg) ^a^	11.31 ± 3.78
PP (mmHg) ^a^	54.87 ± 12.45
LVEDD (mm) ^a^	51.24 ± 4.83
LVESD (mm) ^a^	31.80 ± 4.28
IVSEDD (mm) ^a^	12.81 ± 2.26
PWEDD (mm) ^a^	11.21 ± 2.32
LA (mm) ^a^	42.03 ± 4.66
Ao (mm) ^a^	35.13 ± 3.95
LVEF (%) ^a^	65.67 ± 4.63
LVMI (g/m^2^) ^a^	115.61 ± 41.78
RWT ^a^	0.47 ± 0.09
LVH ^b^	63.4

^a^—values represent mean ± standard deviation, ^b^—values represent percentages, Ao—aortic bulb diameter, IVSEDD—interventricular septum end-diastolic diameter, LA—left atrium diameter, LVEDD—left ventricular end-diastolic diameter, LVEF—left ventricular ejection fraction, LVESD—left ventricular end-systolic diameter, LVH—left ventricular hypertrophy, LVMI—left ventricular mass index, MBP—mean blood pressure, MDBP—mean diastolic blood pressure, MSBP—mean systolic blood pressure, PP—pulse pressure, PWEDD—posterior wall diastolic diameter, RWT—relative wall thickness, VDBP—variability of diastolic blood pressure, VSBP—variability of systolic blood pressure.

**Table 4:** Selenoprotein P, peroxiredoxin-5, renalase, selected parameters of the ABPM and echocardiography in the study subgroups divided based on the criteria of arterial hypertension and obstructive sleep apnoea: A vs. B (diagnosed arterial hypertension vs. without arterial hypertension) and C vs. D (diagnosed obstructive sleep apnoea vs. without obstructive sleep apnoea).

Subgroup	SELENOP (ng/mL) ^a^	Prdx-5 (ng/mL) ^a^	Renalase (ng/mL) ^a^	MBP (mmHg) ^a^	PP (mmHg) ^a^	LA (mm) ^a^	LVEF (%) ^a^	LVH ^b^
A	0.95 ± 0.92	1.27 ± 1.43	159.16 ± 207.19	97.39 ± 11.81	56.98 ± 12.73	44.51 ± 4.39	65.44 ± 4.86	78.0
B	1.87 ± 2.29	1.62 ± 4.63	191.81 ± 209.24	91.78 ± 13.25	52.80 ± 12.37	39.70 ± 4.84	65.83 ± 4.50	53.0
p A-B	<0.05	ns	ns	<0.05	<0.05	<0.05	ns	<0.05
C	1.44 ± 1.95	1.61 ± 4.15	167.37 ± 197.29	95.09 ± 12.98	56.00 ± 12.42	42.46 ± 4.51	65.36 ± 4.88	71.0
D	1.69 ± 1.82	1.96 ± 1.50	212.56 ± 238.79	93.98 ± 11.93	51.44 ± 12.18	40.72 ± 4.93	66.64 ± 3.67	40.0
p C-D	ns	ns	ns	ns	<0.05	ns	ns	<0.05

^a^—values represent mean ± standard deviation, ^b^—values represent percentages, LA—left atrium diameter, LVEF—left ventricular ejection fraction, LVH—left ventricular hypertrophy, MBP—mean blood pressure, PP—pulse pressure.

**Table 5:** Selected parameters of the ABPM and echocardiography in the study subgroups divided based on the criteria of median selenoprotein P, peroxiredoxin-5 and renalase: E vs. F (≥median of selenoprotein P vs. <median of selenoprotein P), G vs. H (≥median of peroxiredoxin-5 vs. <median of peroxiredoxin-5) and I vs. J (≥median of renalase vs. <median of renalase).

Subgroup	MBP (mmHg) ^a^	PP (mmHg) ^a^	LA (mm) ^a^	LVEF (%) ^a^	LVH ^b^
E	91.54 ± 11.07	50.72 ± 9.50	40.12 ± 4.49	66.52 ± 4.22	50.0
F	98.02 ± 13.42	58.94 ± 13.70	43.90 ± 4.05	64.84 ± 4.89	76.5
p E-F	<0.05	<0.05	<0.05	ns	<0.05
G	94.58 ± 12.35	53.82 ± 10.79	42.76 ± 4.85	65.24 ± 4.28	59.2
H	95.03 ± 13.10	55.87 ± 13.87	41.35 ± 4.41	66.08 ± 4.94	67.3
p G-H	ns	ns	ns	ns	ns
I	94.90 ± 12.78	52.51 ± 10.51	40.86 ± 4.85	65.57 ± 4.18	56.9
J	94.72 ± 12.70	57.28 ± 13.86	43.22 ± 4.17	65.78 ± 5.08	70.0
p I-J	ns	<0.05	<0.05	ns	ns

^a^—values represent mean ± standard deviation, ^b^—values represent percentages, LA—left atrium diameter, LVEF—left ventricular ejection fraction, LVH—left ventricular hypertrophy, MBP—mean blood pressure, PP—pulse pressure.

**Table 6:** Selenoprotein P, peroxiredoxin-5, renalase and selected parameters of the echocardiography in the study subgroups divided based on the criteria of selected parameters of the ABPM: K vs. L (≥median of mean blood pressure vs. <median of mean blood pressure) and M vs. N (≥median of pulse pressure vs. <median of pulse pressure).

Subgroup	SELENOP (ng/mL) ^a^	Prdx-5 (ng/mL) ^a^	Renalase (ng/mL) ^a^	LA (mm) ^a^	LVEF (%) ^a^	LVH ^b^
K	1.28 ± 1.66	1.77 ± 5.01	184.68 ± 206.70	40.04 ± 4.34	65.47 ± 5.17	66.7
L	1.73 ± 2.13	1.17 ± 1.32	172.32 ± 211.23	44.02 ± 5.01	65.30 ± 3.66	60.0
p K-L	ns	ns	ns	<0.05	ns	ns
M	0.80 ± 1.04	1.68 ± 4.72	143.45 ± 176.30	43.64 ± 3.98	64.95 ± 5.09	77.6
N	2.45 ± 2.37	1.20 ± 1.31	225.92 ± 238.38	39.86 ± 4.66	66.65 ± 3.75	44.2
p M-N	<0.05	ns	<0.05	<0.05	ns	<0.05

^a^—values represent mean ± standard deviation, ^b^—values represent percentages, LA—left atrium diameter, LVEF—left ventricular ejection fraction, LVH—left ventricular hypertrophy.

**Table 7:** Selenoprotein P, peroxiredoxin-5, renalase and selected parameters of the ABPM in the study subgroups divided based on the criteria of selected parameters of the echocardiography: O vs. P (≥median of left atrium diameter vs. <median of left atrium diameter), R vs. S (≥median of left ventricular ejection fraction vs. <median of left ventricular ejection fraction) and T vs. U (diagnosed left ventricular hypertrophy vs. without left ventricular hypertrophy).

Subgroup	SELENOP (ng/mL) ^a^	Prdx-5 (ng/mL) ^a^	Renalase (ng/mL) ^a^	MBP (mmHg) ^a^	PP (mmHg) ^a^
O	0.85 ± 1.07	1.83 ± 4.89	123.55 ± 172.81	96.06 ± 13.06	57.81 ± 12.24
P	2.23 ± 2.34	1.08 ± 1.40	239.30 ± 227.66	93.43 ± 12.23	51.63 ± 11.99
p O-P	<0.05	ns	<0.05	ns	<0.05
R	1.35 ± 1.59	1.74 ± 5.12	177.66 ± 204.45	95.13 ± 13.71	57.45 ± 13.07
S	1.60 ± 2.08	1.25 ± 1.61	179.34 ± 212.94	94.92 ± 11.04	52.63 ± 11.55
p R-S	ns	ns	ns	ns	<0.05
T	1.02 ± 1.54	1.58 ± 4.47	131.18 ± 157.18	97.21 ± 13.53	57.06 ± 13.29
U	2.33 ± 2.21	1.29 ± 1.57	260.51 ± 257.00	90.67 ± 9.91	51.08 ± 10.37
p T-U	<0.05	ns	<0.05	<0.05	<0.05

^a^—values represent mean ± standard deviation, MBP—mean blood pressure, PP—pulse pressure.

**Table 8:** The results of the correlation analysis in the study group. The table shows statistically significant correlation coefficients.

Tested Parameter	Selenoprotein P (ng/mL)	Peroxiredoxin-5 (ng/mL)	Renalase (ng/mL)
MSBP (mmHg)	−0.33	ns	ns
MDBP (mmHg)	−0.21	ns	ns
MBP (mmHg)	−0.28	ns	ns
VSBP (mmHg)	ns	ns	ns
VDBP (mmHg)	ns	ns	ns
PP (mmHg)	−0.32	ns	−0.22
LVEDD (mm)	ns	ns	ns
LVESD (mm)	ns	ns	ns
IVSEDD (mm)	−0.28	ns	−0.20
PWEDD (mm)	−0.27	ns	ns
LA (mm)	−0.30	ns	−0.22
Ao (mm)	ns	ns	ns
LVEF (%)	ns	ns	ns
LVMI (g/m^2^)	−0.33	ns	ns
RWT	−0.20	ns	ns

Ao—aortic bulb diameter, IVSEDD–interventricular septum end-diastolic diameter, LA—left atrium diameter, LVEDD–left ventricular end-diastolic diameter, LVEF—left ventricular ejection fraction, LVESD—left ventricular end-systolic diameter, LVMI—left ventricular mass index, MBP–mean blood pressure, MDBP—mean diastolic blood pressure, MSBP—mean systolic blood pressure, PP–pulse pressure, PWEDD—posterior wall diastolic diameter, RWT—relative wall thickness, VDBP–variability of diastolic blood pressure, VSBP–variability of systolic blood pressure.

**Table 9:** Results of regression analysis in the study group.

Multivariable Stepwise Backward Regression Analysis			
Model for: PP (mmHg)			
	Rc	SEM of Rc	*p*
intercept	44.801	8.308	< 0.001
age (years)	0.118	0.043	<0.05
BMI (kg/m^2^)	0.355	0.133	<0.05
antihypertensives drugs	−3.757	1.250	<0.01
SELENOP (ng/mL)	−1.876	0.636	<0.01
renalase (ng/mL)	−0.011	0.004	<0.05
*p* < 0.01			
Multivariable Stepwise Backward Regression Analysis			
Model for: LA (mm)			
	Rc	SEM of Rc	*p*
intercept	38.929	3.254	<0.001
age (years)	0.045	0.022	<0.05
BMI (kg/m^2^)	0.004	0.001	<0.01
male gender	1.762	0.792	<0.05
PP (mmHg)	0.111	0.037	<0.01
*p* < 0.01			
Logistic Regression Analysis			
Model for: Probability of LVH			
	Rc	SEM of Rc	*p*
BMI (kg/m^2^)	0.079	0.021	<0.01
antihypertensives drugs	−1.448	0.321	<0.01
OSA	1.210	0.514	<0.05
PP (mmHg)	0.043	0.020	<0.05
*p* < 0.05			

BMI—body mass index, LA—left atrium diameter, LVH—left ventricular hypertrophy, OSA—obstructive sleep apnoea, PP—pulse pressure, Rc—regression coefficient, SEM—standard error of mean.

## Data Availability

The data presented in this study are available on request from the corresponding author. The data are not publicly available due to privacy.
